# Multi-organ dysfunction secondary to severe wasp envenomation

**DOI:** 10.1186/s12245-015-0054-7

**Published:** 2015-03-12

**Authors:** Abraham M Ittyachen, Shanavas Abdulla, Rifzana Fathima Anwarsha, Bhavya S Kumar

**Affiliations:** Malankara Orthodox Syrian Church Medical College and Hospital, Ernakulam District, Kolenchery, Kerala State 682311 India

**Keywords:** Wasp sting, Allergy, Multi-organ dysfunction

## Abstract

Wasp sting is not an uncommon incident. Around 56% to 94% of the population is stung at least once in their lifetime by a member of the order Hymenoptera which includes wasps, bees, and ants. The response to a wasp sting may vary from mild local reaction to severe systemic and anaphylactic reactions. The clinical picture and mortality rate tend to be more severe in adults compared to children. We present a 32-year-old agricultural worker who was bitten by multiple wasps while on a coconut tree. In spite of the heavy load of venom due to the multiple bites, the patient did not develop anaphylaxis. However, a delayed reaction did occur within 48 h in the form of severe multi-organ dysfunction. There was significant improvement by around 2 weeks; but it took another 6 months for the serum creatinine to normalize. This case highlights the occupational risk of Hymenoptera envenomation, the life-threatening complications that may follow and which may even be delayed as was the case with this patient, and the value of emergency care and intensive management which can result in a favorable clinical outcome.

## Background

Wasp sting is not an uncommon incident. Wasps together with bees and ants belong to the order Hymenoptera. Around 56% to 94% of the population is stung by a member of this order at least once in their lifetime [1]. The response to Hymenoptera stings are classified as normal local reactions, large local reactions, systemic anaphylactic reactions, systemic toxic reactions, and unusual reactions [2,3]. The most frequently observed are large local and systemic anaphylactic reactions [2]. In children, around 60% of systemic sting reactions are mild, whereas in adults, systemic reactions tend to be severe in about 70% [4]. Also, the fatality rate is higher in elderly patients than that in children and young adults [4,5]. Herein, we present a young male who had multi-organ dysfunction secondary to multiple wasp stings.

## Case presentation

A 32-year-old male presented to the emergency department (ED) of our hospital with breathing difficulty and decreased urine output. One day prior to the arrival in our hospital, this patient had been bitten by multiple wasps (exact species could not be identified) while climbing a coconut tree. He had developed breathing difficulty and generalized swelling immediately after this incident. He was taken to a nearby hospital where he was treated in the emergency unit and send home the same day as he felt ‘better’. The next day, he noticed a decrease in his urine output and progressive shortness of breath and hence was referred to our hospital.

This was a young male who was engaged in agricultural work. He had a history of bronchial asthma since childhood and used to take albuterol inhalers occasionally. There was no history of severe wheezing until now. He neither smoked nor took alcohol and also denied taking any illicit drugs.

**Figure 1:**
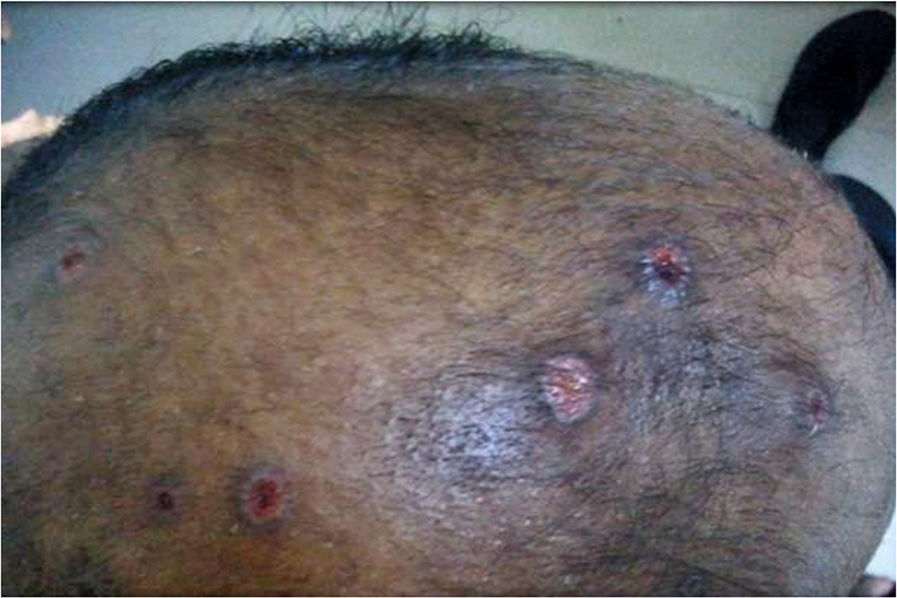
**Ulcerations over the scalp.**

**Figure 2:**
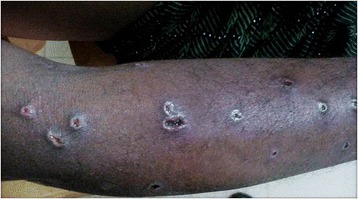
**Ulcerations over the upper limb.**

At arrival in the ED, he was conscious and oriented. He appeared jaundiced with multiple swellings predominantly over the trunk, upper limbs, and head (these swellings later developed mild ulcerations) (Figures 1 and 2). There was mild tachycardia (110 beats/min) and tachypnea (24 times/min). Oxygen saturation was 92% on room air, and blood pressure was recorded as 140/90 mm of Hg in the right upper limb. The rest of systemic examination was unremarkable.

Based on a clinical suspicion of multi-organ dysfunction, he was evaluated as such. His laboratory parameters were significant for severe renal failure, hemolysis, rhabdomyolysis, and liver dysfunction. His blood urea was 126 mg/dL (N: 20 to 40 mg/dL) and serum creatinine was 6.1 mg/dL (N: 0.5 to 1.4 mg/dL). His liver enzymes were also significantly raised: alanine transaminase (ALT) 7128 IU/L (N: 5 to 35 IU/L) and aspartate transaminase (AST) 13,985 IU/L (N: 8 to 40 IU/L). Coagulation parameters were mildly prolonged: prothrombin time 27.2 s (test) and 11.3 s (control) and international normalized ratio (INR) 2.47. Though the patient had significant elevation in liver enzymes, he did not have any clinical features of liver cell failure (hepatic encephalopathy). The disproportionate rise in liver enzymes together with increased bilirubin levels (total bilirubin 22.3 mg/dL N: 0.2 to 1.2 mg/dL) and a prolonged prothrombin time was presumably due to rhabdomyolysis and hemolysis/disseminated intravascular coagulation (DIC). A significant rise in the level of lactate dehydrogenase (LDH) corroborated this finding (LDH: 76,100 IU/L N: 164 to 412 IU/L). Also, his hemogram was deranged with the presence of anemia (hemoglobin: 8 gm/dL) and thrombocytopenia (platelets: 25,000/mm^3^). His creatine phosphokinase (CPK) levels were also markedly elevated - rhabdomyolysis (CPK: 35,029 IU/L N: 0 to 225 IU/L). However, urine did not show any myoglobinuria. Viral markers for hepatitis B, hepatitis C, hepatitis A, and HIV were negative. Being an area endemic for leptospirosis, the same was also ruled out.

**Figure 3:**
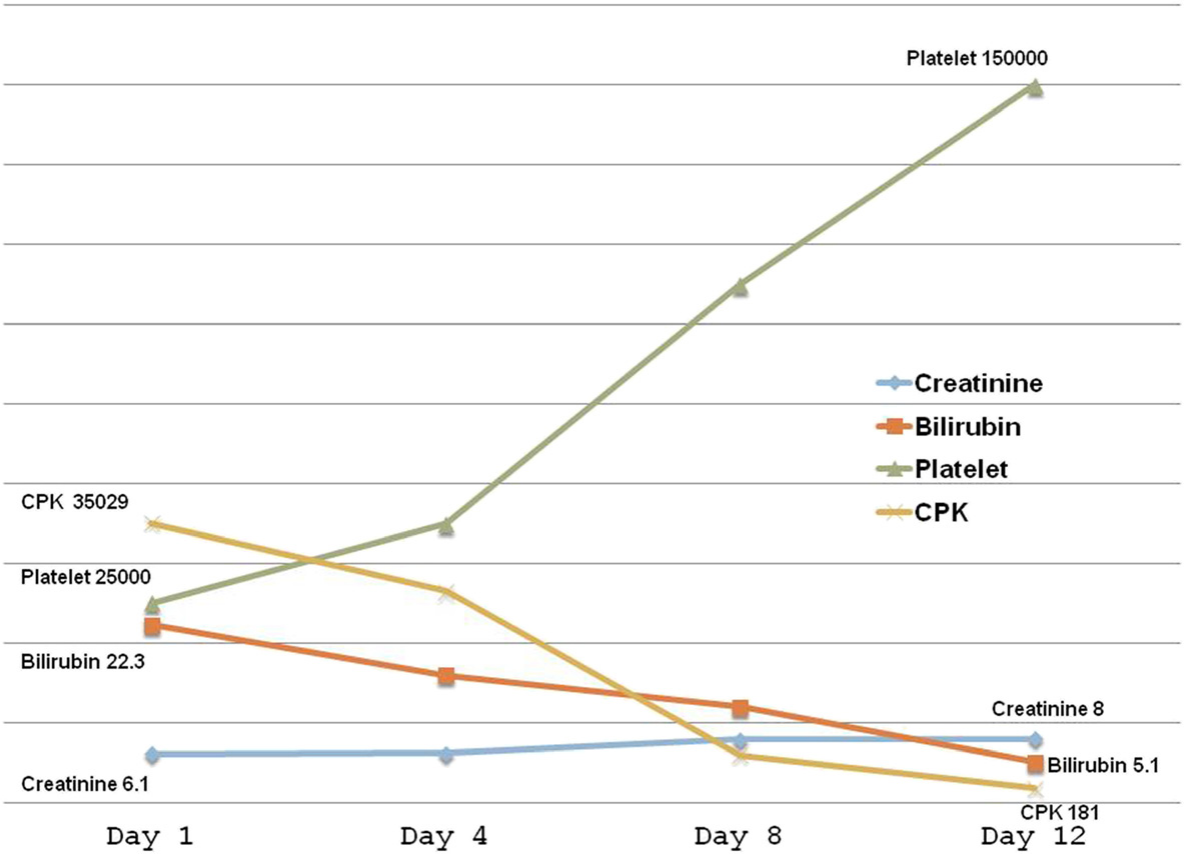
**Laboratory parameters.** Note that all the parameters except creatinine had improved by about 2 weeks. It took another 6 months for the creatinine to normalize.

Patient was managed initially in the intensive care unit (ICU). He received intravenous methylprednisolone and antihistamines (pheniramine maleate) for the first 24 h. He continued to be anuric for the first 14 days and in all received 12 sessions of hemodialysis till the urine output improved and uremic symptoms subsided. He was also transfused 6 units of fresh frozen plasma (FFP) and 4 units of platelet concentrate. The rest of the treatment included intravenous fluids, prophylactic antibiotics, oxygen, and symptomatic measures. By about 2 weeks, his laboratory parameters started improving (Figure 3). By the end of the third week, he was discharged successfully. Though urine output had been established, it took another 6 months for his serum creatinine value to normalize. Throughout his hospital stay, the patient was hemodynamically stable and also did not require any assisted ventilation.

## Discussion

Hymenoptera sting happens to be an occupational hazard in many parts of the world [6] as was the case of this patient. The risk for systemic reaction is increased if preceded by a sting within the last 2 months even if the first sting is well tolerated [7]. However, our patient did not reveal a prior history of envenomation despite the fact that he had an occupational risk. Though the risk for systemic reaction is described to be greater in a bee venom-sensitized patient compared with those sensitized to wasp venom [8], our patient was an exception.

The toxic compounds described in wasp venom are phospholipase A1, hyaluronidase, and antigen 5 [9,10]. Other allergens include active peptides like melittin, amines like histamine and serotonin, and kinins, apamine, and acetylcholine. Together, they are responsible for the myriad presentation of wasp sting. The more severe systemic complications include renal (acute renal failure, nephrotic syndrome, and renal tubular acidosis) [11,12], cardiac (myocarditis, myocardial infarction, and arrhythmias) [13,14], hepatic (centrilobular necrosis and pericholangitis) [15,16], neurological (stroke, Guillian-Barre syndrome, and acute encephalopathy) [17,18], hematological (hemolysis, DIC, and thrombocytopenia) [19-21], and vasculitis [22]. Our patient had several of these systemic reactions. However, in spite of the heavy load of venom from the multiple bites, this patient did not develop the classic feature of severe anaphylaxis, specifically, anaphylactic shock.

For patients who have an occupational risk for insect envenomation, immunotherapy may be a remedial measure. However, the clinical effectiveness may be questionable [23] not to mention the cost involved, especially for poor communities. Monoclonal antibody as an adjuvant to immunotherapy to increase the effectiveness of such therapy [24] may be a future option but the issue of cost still remains.

## Conclusions

Although the management of any envenomation whether it produces immediate anaphylaxis or late complications is challenging, the role of emergency care and intensive management leading to a favorable outcome cannot be overemphasized.

Emergency physicians should be aware of the risk of hymenoptera envenomation in certain occupations and the life-threatening complications that may follow and which may even be delayed as was the case with this patient.

## Consent

Written informed consent was obtained from the patient for publication of this case report and any accompanying images.

## Abbreviations

ED: Emergency department
ALT: Alanine transaminase
AST: Aspartate transaminase
INR: International normalized ratio
DIC: Disseminated intravascular coagulation
LDH: Lactate dehydrogenase
CPK: Creatine phosphokinase
HIV: Human immunodeficiency virus
ICU: Intensive care unit
FFP: Fresh frozen plasma
